# The impact of county-level factors on meaningful use of electronic health records (EHRs) among primary care providers

**DOI:** 10.1371/journal.pone.0295435

**Published:** 2024-01-25

**Authors:** Pierre K. Alexandre, Judith P. Monestime, Kessie Alexandre

**Affiliations:** 1 Health Administration Program, Department of Management, College of Business, Florida Atlantic University, Boca Raton, Florida, United States of America; 2 Department of Geography, University of Washington, Seattle, Washington, United States of America; University of South-Eastern Norway: Universitetet i Sorost-Norge, NORWAY

## Abstract

This study examines the impact of county-level factors on “meaningful use” (MU) of electronic health records (EHRs) for 8415 primary care providers (PCPs) that enrolled in the Florida Medicaid EHR Incentive Program through adopting, improving, or upgrading (AIU) a certified EHR technology. PCPs received incentive payments at enrollment and if they used their EHRs in meaningful ways; ways that benefit patients and providers alike they received additional payments. We conducted a retrospective cohort study of these providers over the 2011–2018 period while linking their records to other state data. We used the core constructs of the resource dependence theory (RDT), a well-established organization theory in business management, to operationalize the county-level variables. These variables were rurality, poverty, educational attainment, managed care penetration, changes in population, and number of PCPs per capita. The unit of analysis was provider-years. For practical and computational purposes, all the county variables were dichotomized. We used analysis of variance (ANOVA) to test for differences in MU attestation rates across each county variable. Odds ratios and corresponding 95% confidence intervals were derived from pooled logistic regressions using generalized estimated equations (GEE) with the binomial family and logit link functions. Clustered standard errors were used. Approximately 42% of these providers attested to MU after receiving first-year incentives. Rurality and poverty were significantly associated with MU. To some degree, managed care penetration, change in population size, and number of PCPs per capita were also associated with MU. Policy makers and healthcare managers should not ignore the contribution of county-level factors in the diffusion of EHRs among physician practices. These county-level findings provide important insights about EHR diffusion in places where traditionally underserved populations live. This county-perspective is particularly important because of the potential for health IT to enable public health monitoring and population health management that might benefit individuals beyond the patients treated by the Medicaid providers.

## Introduction

The diffusion of a technology encompasses both the decision to adopt a technology (inter-firm diffusion) and the extent at which adopting firms use the capabilities of the technology (intra-firm diffusion) [1]. But studies on the diffusion of health information technology (health IT) among primary care providers (PCPs) typically focus on whether these providers adopt electronic health record (EHR) systems rather than on the use of these systems’ capabilities across providers [2, 3]. There exist approximately 250,000 physician practices in the United States [4]. They typically use a wide range of EHR systems, from basic to advanced EHR systems [3]. The basic EHR systems replace the traditional medical charts and collect demographic and clinical data about the patient, including medical history, medications taken, and laboratory results [2, 3]. The advanced EHR systems incorporate computerized provider order entry (CPOE) capabilities that allow providers to electronically enter medical orders for patient medications and diagnostic tests [2, 3]. They also allow providers to write clinical notes and provide treatment guidelines [2, 3]. Hence, the advanced EHR systems can both complement and substitute labor for medical practices, involving clinical decision making and complex communications with pharmacies, laboratories, and the patients [5]. This combination of technological and organizational capabilities is commonly referred to as “Meaningful Use” (MU) of EHRs [5].

In an effort to improve the quality and effectiveness of healthcare through the expanded use of EHRs in the United States, the Health Information Technology for Economic and Clinical Health (HITECH) Act, enacted as part of the 2009 American Recovery and Reinvestment Act (ARRA), was signed into law [6]. From 2011 to 2021, using funds available from the HITECH Act, the Centers for Medicare and Medicaid Services (CMS) funded the Medicaid EHR Incentive Program, later renamed the Medicaid Promoting Interoperability (MPI) program. In addition to adopting a certified EHR technology, this program required that PCPs use this technology in a “meaningful” way to ensure that health information is shared and exchanged to improve patient care [7]. At the end of 2010, CMS issued the criteria relating to how eligible PCPs can demonstrate “meaningful use” of EHR technology to qualify for incentive payments [8]. These criteria fall into four general categories: vocabulary, content exchange, privacy and security, and transport. More specifically, standards were set forth with regards to how PCPs should (a) record clinical summaries and prescriptions; (b) describe clinical procedures, laboratory tests, and medications; and (b) secure the transmission of information using the internet [8]. These standards included fifteen “core set” objectives that all PCPs must achieve and 10 “menu set” objectives, of which PCPs must select 5 [8, 9]. Overall, the goals were to improve safety, quality, and efficiency of healthcare and reduce health disparities, engage patients and families in their care, improve population and public health, protect privacy and security of health information, and reduce healthcare costs [8, 9]. The MPI program was voluntary for state Medicaid agencies; but all the 50 states and the District of Columbia participated. The federal government covered about 90 percent of the cost [8].

Eligible providers included physicians, nurse practitioners, certified nurse-midwives, dentists, and physician assistants. To qualify they must meet a minimum of 30% Medicaid encounter volume threshold over a 90-day period, but pediatricians could qualify with a 20% Medicaid encounter volume. Additional requirements included being in active status, having no outstanding state or federal sanctions, and not being in a hospital setting. Providers must first register at the CMS—Registration and Attestations System (R&A). Once registered, they can apply online using the Medical Assistance Provider Incentive Repository (MAPIR) system. Each provider then received an incentive payment of $21,250 for committing to "adopt, implement or upgrade" (AIU) a certified EHR system. In practice, “adoption” consists of acquiring or purchasing a certified EHR system; “implementation” consists of installing or starting to use a certified EHR system; and “upgrading” consists of improving an existing EHR to a certified EHR system. Enrollment through AIU phases ended in 2016.

Providers received a second incentive payment of $8500 for attesting to MU; but pediatricians that enrolled with a 20–29% Medicaid encounter volume received $4250 [8, 9]. Payments for attesting to MU for the first time after AIU enrollment ended in 2018. Pre-payment controls were built into the MAPIR system to detect inaccuracies in eligibility, reporting, and payments. Provider audits that targeted suspicious and anomalous data and random audits were performed to ensure that incentive payments were made to providers that met all program requirements. Providers were not required to participate in subsequent or consecutive years. It is worth noting that eligible providers could also enroll into the MPI program simply by attesting to MU of EHRs in a 90-day reporting period, but these providers were excluded from this study. Further details on the program can be found elsewhere [8, 9].

The present study used data from the Florida—Medicaid Promoting Interoperability (FL-MPI) program to examine the impact of county-level factors on MU attestation rates among PCPs after receiving the first-year incentive payments. Its contribution to the literature is significant. Overall, the broad question posed was: which environmental factors influence the widespread use of advanced EHR functions by PCPs? The corresponding policy implications are whether tools can be developed to overcome existing county-level disparities. Nearly 9 out of 10 physician practices in the U.S. have adopted a basic EHR system [10]. But the rate of MU attestations has stalled among Medicaid providers, providing evidence of an emerging advanced use “digital divide”, contrary to the conclusion reached for hospitals [10–12]. Research indicates that the use of advanced EHR functions holds promise to address socioeconomic disparities for underserved populations [13, 14]. As health disparities are often geographically linked, it is important to obtain contextual insights about MU attestation rates among Medicaid providers.

## Materials and methods

### Theoretical framework

The theoretical framework for this study is the resource dependence theory (RDT), a well-developed organization theory in business management [15–17]. It offers relevant concepts to understand the diffusion of EHRs by identifying the importance of environmental factors [18–20]. RDT dictates that providers will do what they can to secure resources from their environments through a variety of actions or exchanges to reach their goals of attesting to MU of EHRs [17].

The three core constructs of RDT are environmental munificence, dynamism, and complexity [17]. Environmental munificence is concerned with the availability and accessibility of environmental resources [21, 22]. In this instance, it is tied to the availability of financial resources in the providers’ markets as they seek financial resources in the forms of direct payments from the patients and reimbursements from public and private insurers [18]. For example, providers in wealthier environments that cater to a more affluent and educated customer base might find it more beneficial to use the advanced functions of their EHR systems (patient portal; e-prescribe, etc.) to appeal to potential clients, typically more selective in their healthcare services [23]. The second core construct, environmental dynamism, reflects the rate of change in the environments, which increases the providers’ level of uncertainty [17, 22, 24]. Providers in uncertain markets must adapt quickly to survive as the need to strategize to secure resources from these markets becomes more pressuring [22, 24]. The last core construct, environmental complexity, reflects the level of competition in the markets [22, 25, 26]. In the face of competition, providers must compete for the scarce resources as well as secure their share of patients [18]. For example, they might find it beneficial to adopt a health technology in areas of high competition as they need to appeal to the patient population [18, 23].

**Fig 1:**
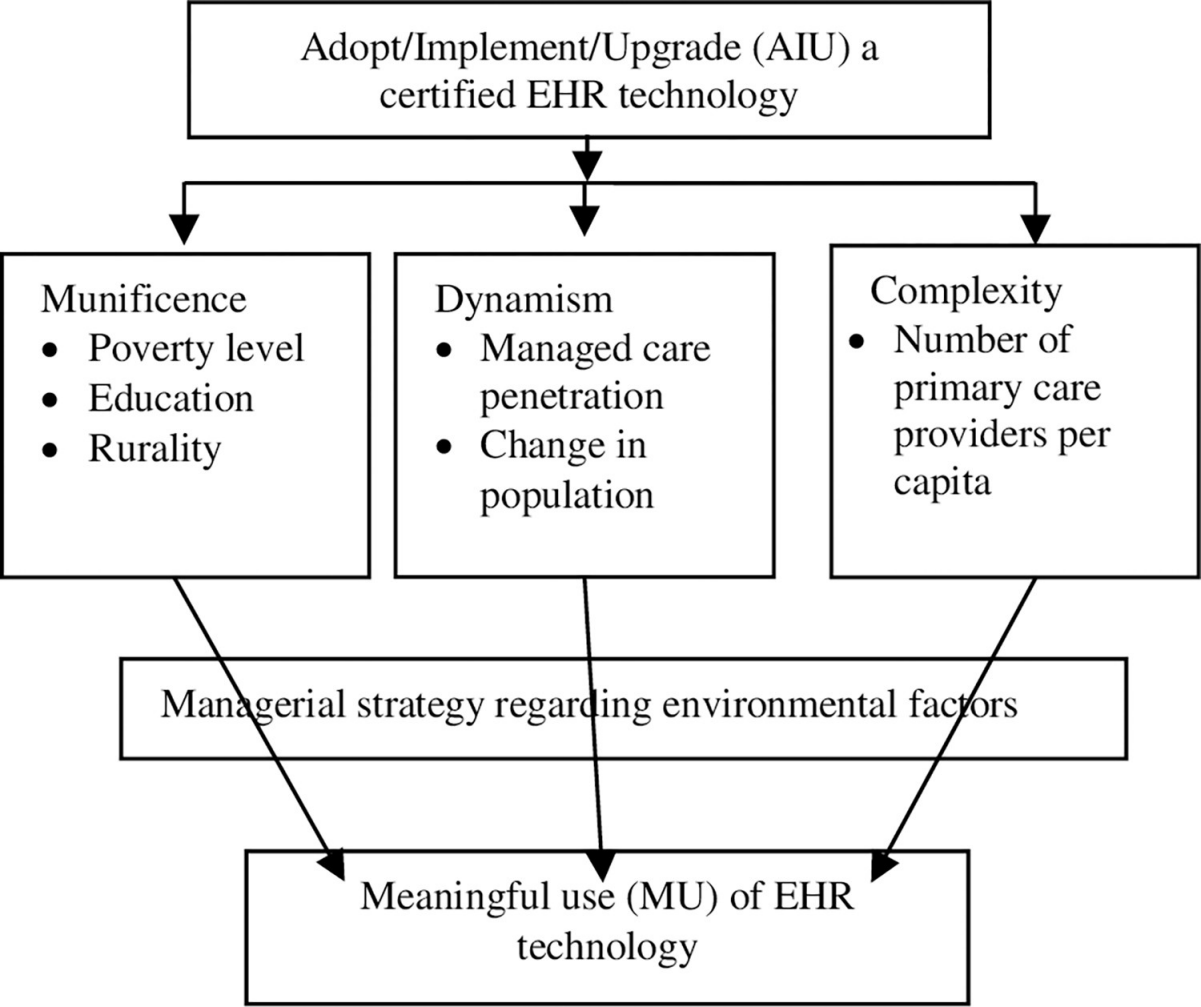
Conceptual model: Resource Dependence Theory (RDT).

Fig 1 depicts the theoretical model of this study. It is adapted from Kissam et al. [27] and Menachemi et al. [19] and incorporates the variables that characterize the core constructs of RDT.

Although studies that employ RDT in examining healthcare providers are increasingly common, there exists little consensus on how to operationalize the market environment in the context of healthcare. With the exception of education, all the variables used for the RDT constructs in Fig 1 above were selected using a systematic review conducted by Yeager and colleagues who reviewed twenty studies that relied on aspects of RDT to examine the healthcare market [28]. The authors summarized how the environment was measured in those studies and provided a list of variables that operationalized the RDT constructs [28]. Educational attainment, typically used in economic studies as a proxy for human capital and skills available in a market [1, 3], has also been used to operationalize environmental munificence in healthcare [27]. The characteristics of the providers are not themselves part of RDT although they might enable or hinder the providers’ ability to attest to MU [18, 29, 30]. They are thus considered as controls in this study.

### Data sources

The main database for this study was the Provider Participation Database for the FL-MPI program [31]. It supplied data on each provider’s unique 10-digit national provider identification (NPI), enrollment year, incentives payment amounts and dates, AIU phases, providers’ specialties and zip codes, and practice types. The Florida Office of Insurance Regulation (FOIR) database provided data on managed care enrollments [32]. Several measures came from the Area Health Resource Files (AHRF), a national database that provides current as well as historic data for more than 6,000 variables for each of the U.S. counties [33]. The data source years included vary based on data availability but typically cover the previous 10 years. The AHRF data include information on healthcare professions, population characteristics, economics, healthcare utilization and expenditures, and health facilities. The basic file contains geographic codes and descriptors that enable it to be linked to many other files. We used the NPI and Florida county identifiers to link data from the above databases and construct the analysis sample.

### Meaningful use (MU) attestations of EHRs

At enrollment, providers applied for the first-year incentive payment (Payment Year 1) and attested to one of the AIU phases as discussed earlier. They received a second payment (Payment Year 2) after attesting to meaningful use of EHRs. The outcome variable (MU) thus indicated whether a provider attested to MU during the study period. It was equal to 1 if a second payment was received and 0 otherwise.

### County-level variables

We selected county variables that represented the three dimensions of the environment as shown in Fig 1. A county’s munificence was operationalized using three variables: rurality, poverty rate, and educational attainment [18, 21, 25]. We categorized the counties as urban and rural (100 persons or less per square mile) using a Florida rurality map based on the 2010 census [34]. Poverty rate was measured as the percentage of the county’s population that lived in poverty defined as not having enough money to meet basic needs including food, clothing and shelter. Educational attainment was measured as the percentage of the county’s population aged 25 years and older with a high school education. To represent environmental dynamism, we estimated the percentage of the county’s population covered by a health maintenance organization (HMO) [32] and the percentage change in each county’s population during the study period [19, 35]. Lastly, environmental complexity was measured by the number of nonfederal PCPs practicing in the county. It was expressed as the number of PCPs per 10,000 population. Except for the HMO enrollment data, all the county rates were estimated using data from the AHRF.

### Control variables

The selection of the control variables was based on previous studies on EHR diffusion. Our analysis controlled for practices’ characteristics, including providers’ specialty and practice type. The literature indicates that provider’s specialty is an important factor in optimizing the use of EHRs [30]. Indicators for providers’ specialty included being a physician, certified nurse midwife, nurse practitioner, dentist, pediatrician, or physician assistant. We used the NPI to obtain information on practice ownership, that is, whether the practice was an independent practice or part of a group practice. Practice type might affect the extent of use of EHR capabilities because of differences in the complexity of the decision structure and other human and economic performances [36, 37]. For example, group practices are more likely to have greater access to financial and managerial resources and may experience economies of scale by sharing resources such as administrative staff [27]. We also controlled for EHR use at enrollment using AIU phase data to reflect the providers’ sophistication with computer use [30, 38–40]. We assumed that practices with a more enhanced EHR technology at enrollment might have a greater ability to use their EHR capabilities to achieve meaningful use of EHRs [38]. Lastly, county and year dummies were included in the regression analysis.

## Analytical approach

We conducted a 2011–2018 retrospective cohort study of 8415 providers that participated in the FL-MPI program. We narrowed our analysis to the counties that had a minimum of 10 providers to obtain enough grids for the county-level analysis [41, 42]. This reduced the number of counties from 67 to 53 counties. Because the dataset included only the dates the providers received payments for having attested to MU, it was not possible to determine the exact years of MU attestations. Hence, the unit of analysis was provider-years. Our sample included 2622, 2035, 1565, 777, 718, 698 providers that enrolled into the program, from 2011 to 2016, respectively. All the county ordinal variables were averaged over the study period. All data accessed complied with data protection regulation.

We calculated and presented descriptive statistics to characterize our sample. Next, we used one-way analysis of variance (ANOVA) to test for differences in MU attestation rates across each county variable. We then used pooled logistic regressions with generalized estimated equations (GEE) and the binomial family and logit link functions to examine the associations between MU attestations and the county-level variables, controlling for AIU phases and practices’ characteristics [43–45]. County and year dummies were also included. Using the delta methods, we estimated clustered standard errors to adjust for the non-independence of observations within counties [46–48]. In view of the large number of explanatory variables, we calculated the vector of variance inflator factors (VIF) to check for possible multicollinearity. We found that the highest VIF was below 5 and the mean VIF was 2.21, indicating that correlations between the covariates were relatively acceptable and that multi-collinearity was not a serious problem [48]. Odds ratios and corresponding 95% confidence intervals were derived using Stata 16 [49].

## Results

Table 1 displays the descriptive statistics for the characteristics of the sample. The results indicate that, among the 8415 provider-years, physicians represented the largest group (52.1%), followed by nurse practitioners (23.6%), pediatricians (13.9%), dentists (8.1%), certified nurse-midwives (1.9%) and physician assistants (0.4%). Group practices were more common (63.4%) than solo practices (36.6%). For the AIU phases, we found that 36.6% of the providers in the sample adopted a certified EHR system; 28.5% implemented a certified EHR system; and 34.9% upgraded to a certified EHR system.

**Table 1:** Descriptive statistics for the sample (*N* = 8415 provider-years, 2011–2018).

Characteristic	Overall N (%)
Specialty	
Physician	4380(52.1)
Pediatrician	1172(13.9)
Dentist	680(8.1)
Nurse Practitioner	1985(23.6)
Physician Assistant	37(0.4)
Certified Nurse- Midwife	161(1.9)
Practice Type	
Solo	3080(36.6)
Group	5335(63.4)
AIU Phases^a^	
Adopt	3079(36.6)
Implement	2396(28.5)
Upgrade	2940(34.9)

^a^AIU = adopt, implement, or upgrade a certified EHR technology

Table 2 presents MU attestation rates for the overall program and by enrollment year. On the average, 41.9% of the sample achieved MU after receiving the first-year incentive. The result indicates notable decrease in MU attestation rates over time. More specifically, MU attestation rates decreased from 56.7% among the providers that enrolled in 2011 to 19.2% among those enrolled in 2016.

**Table 2:** Meaningful use (MU) attestations rates (*N* = 8415 provider-years).

Yes (N = 3525 provider-years)	41.9%
No (N = 4890 provider-years)	58.1%
Enrollment year	
2011	56.7%
2012	49.6%
2013	30.2%
2014	27.9%
2015	27.4%
2016	19.2%

For practical and computational purposes, we categorized counties as poor counties (poverty rates ≥20%) and non-poor counties (poverty rates < 20%) [50]. The other county rates were converted into three dichotomous variables corresponding to terciles (e.g., Q_1_- tercile group that includes counties with the lowest rates was referred to as “low group”; Q_3_-tercile group with the highest rates was the “high group”; and Q_2_-tercile group was the “medium group”). The results for the bivariate analysis are presented in Table 3. They were all significant at p < .01, indicating that the MU attestation rates between the groups were not all equal. Namely, rural counties, non-poor counties, counties with higher high-school graduation rates, counties with lower HMO penetration rates, counties with larger changes in population, and counties with a lower number of PCPs per capita had higher MU attestation rates.

**Table 3:** Unadjusted MU attestation rates of EHRs by county-level characteristics.

Environmental characteristics	MU attestation rates (%)	p-value
*Rurality*		<0.001
Rural	63.2	
Urban	44.7	
*Poverty*		<0.001
Poor counties	50.1	
Non-poor counties	51.4	
*Persons 25 years and older with a high school education*		<0.001
Q1(20.1–58.8%)	38.7	
Q2 (58.9–63.7%)	49.1	
Q3 (63.8–97.9%)	49.3	
*HMO penetration*		<0.001
Q1 (5.5–15.4%)	55.6	
Q2 (15.5–25.5%)	38.6	
Q3 (25.6–54.1%)	40.7	
*Change in population*		<0.01
Q1(-4.2–1.0%)	46.2	
Q2 (1.1–1.7%)	41.6	
Q3 (1.8–6.8%)	49.3	
*Number of PCPs per 10*,*000 population*		<0.01
Q1(1.7–7.2)	53.2	
Q2 (7.3–7.8)	46.0	
Q3 (7.9–8.1)	36.3	

Table 4 presents the multivariate results for the relationships between county variables and MU attestations. They indicate that providers located in rural counties were 80 percent more likely to attest to MU than providers in urban counties: OR = 1.80; 95% CI [1.30 2.50]. Providers located in poor counties were 32 percent less likely to attest to MU compared to providers in non-poor counties: OR = 0.68; 95% CI [0.55 0.84]. Providers located in counties with high HMO penetration rates were 34 percent less likely to attest to MU than providers in counties with low HMO penetration rates: OR = 0.66, 95% CI [0.57 0.77]. We also found that providers located in counties with moderate changes in their populations over the study period were 27% less likely to attest to MU compared to counties with lower changes in their populations: OR = 0.73, 95% CI [0.63 0.86]. Finally, the results indicate that providers located in counties with a medium number of PCPs per capita were 45 percent more likely to attest to MU compared to providers located in counties with a lower number of PCPs per capita: OR = 1.46, 95% CI [1.24 1.74].

**Table 4:** Multivariate relationships between county-level factors and meaning use (MU) attestations of EHRs^a^.

County-level variables	OR	95%CI
*Geographical location*		
Rural (ref: urban)	1.80**	(1.30–2.50)
*Poverty rate*		
Poor counties (ref: non-poor counties)	0.68**	(0.55–0.84)
*Percent persons 25 years and older with at least a high school education* ^b^		
Q_2_: “medium” group	1.02	(0.84–1.25)
Q_3_: “high” group	1.01	(0.81–1.24)
*HMO penetration rate* ^b^		
Q_2_: “medium” group	0.80**	(0.71–1.16)
Q_3_: “high” group	0.66**	(0.57–0.77)
*Percent change in population* ^b^		
Q_2_: “medium” group	0.73**	(0.63–0.86)
Q_3_: “high” group	0.98	(0.85–1.14)
*Number of primary care providers per 10*,*000 population*^b^		
Q2: “medium” group	1.46**	(1.24–1.74)
Q3: “high” group	0.91	(0.78–1.14)

^a^Regression analysis also controlled for provider specialty (physician, dentist, nurse practitioner, pediatrician, physician assistant), practice type (solo vs. group), EHR phases (adopt, implement or upgrade (AIU) EHRs), and county and year dummies. Clustered standard errors were used to calculate confidence intervals.

^b^Q_1_: first tercile (low group) is the reference group.

* Significant at P<0.05

** Significant at p<0.01

Most of the control variables (not shown on Table 4) were significant in the multivariate logistic regressions. More specifically, compared to physicians, pediatricians were more likely to attest to MU while dentists and nurse practitioners were less likely to attest to MU. Group practices were more likely to attest to MU than solo practices. Finally, we found that providers that implemented or upgraded EHR systems were more likely to attest to MU than those that adopted EHR systems.

To check the robustness of the results, we use the approach used by Monestime et al. [30]. We divided the participants into early participants and late participants, which were subsets of providers who enrolled in 2011–2012 and in 2013–2016, respectively. The results of the logistic regressions remained robust in all the models.

## Discussion

While unexpedted, the findings that providers in rural counties had higher MU attestation rates than providers in urban counties were also found in more recent studies [51, 52]. A plausible explanation is the presence of regional extension centers (RECs) in the rural counties [51]. The REC program was established by the HITECH Act and administered through the office of National Coordinator (ONC) for Health Information Technology. The program included 62 grantee organizations throughout the U.S. that promoted EHR adoption and the optimal use of EHR technology through outreach and the provision of technical assistance [53]. Florida was among the 3 states with the highest number of RECs (4) granted by the ONC [53]. The REC program prioritized small primary care practices in rural areas [53, 54]. Previous research showed that these providers had lower rates of EHR adoption than larger organizations and that they generally lacked resources to adopt and maintain EHRs [55]. The REC program was found to be very successful in engaging providers in rural areas [6, 51, 56].

The findings that a county’s poverty rate is negatively associated with MU of EHRs are consistent with those of most previous studies [57, 58]. They suggests that PCPs in these counties might face challenges to EHR diffusion related to tight operating budgets and limited capacity for integrating EHR training into the workflow [57]. But it is to note that other researchers found mixed results [59, 60]. The results also indicate that HMO penetration rate was negatively associated MU attestations. While others found mixed results [61, 62], this finding is consistent with another study conducted among a large number of physicians in Florida [63]. It is also congruent with the medical technology literature indicating that higher managed care penetrations in geographically defined healthcare markets are associated with lower adoption of new technologies [61, 64]. We also found that moderate changes in a county’s population size was associated with lower MU attestation rates. This finding is consistent with other studies indicating that uncertainty delayed the decision to pursue expensive strategies [22, 65]. Finally, we found that a medium or moderate increase in the number of physicians per capita (10,000) increased the probability of attesting to MU. This is in line with other studies in both healthcare and other industries that suggest that in the face of competition, organizations can adopt strategic decision making, become more innovative, and create competitive advantage [63, 66–68]. Hence, in competitive markets, physicians might use their EHRs to brand their services as high quality and high technology to facilitate referrals [23].

Florida ranks among the states with the highest number of Medicaid recipients (over 6.7 million recipients as of 2023) [69]. It is the third-largest state by population size (22.2 million in 2022) [70]. Florida in 2022 was 77.05% White, 17.02% Black, and 5.93% other [70]. Nearly half (45%) of working Floridians are living in or near poverty [69]. As of 2022, 30 of the Florida’s 67 counties were qualified as rural counties with about 750,000 residents [69]. But more than 1 million persons live in the rural portions of Florida’s 37 urban counties [69]. Hence, Florida’s varied socioeconomic status provides an ideal setting for contextual insights about meaningful use of EHRs among Medicaid providers that serve traditionally underserved communities.

Meaningful use of EHRs requires EHR capabilities that include, among other things, patient portals, patient-monitoring system, and computerized provider order entry (CPOE) as discussed earlier. Leveraging advanced use, EHRs hold great promise to improve poor health outcomes and health equity [9, 10, 71]. The Medicaid Promoting Interoperability Program has greatly contributed to the uptake of technology in clinical care practices in the U.S., as nearly 9 out of 10 offices have adopted a basic EHR system [10]. However, the rate of MU of EHRs has stalled among Medicaid providers [10]. In Florida specifically the majority of Medicaid providers do not use the EHR system to their full capabilities [30]. Our study provides critical insights into the structural barriers (regional, local, economic, and resource-related issues) that hinder the optimal diffusion of health IT for low-resourced providers [71].

Our results might provide useful information to other initiatives to increase EHR diffusion in underserved communities. For example, the Health Resource and Services Administration is supporting several initiatives of EHR diffusion in community health centers and rural health clinics, which are important safety net providers of care for traditionally underserved populations [10]. Our study can provide insights to these initiatives and the like across otherwise similar areas. These findings can also provide insights into the larger field of health informatics regarding factors that can influence an unintended “digital divide” for providers and healthcare institutions in medically underserved communities and regions [13]. This study has limitations related to the datasets used. First, the data are from the state of Florida and, as a result, the generalizability to other states should be done with caution. Second, it was not possible to determine the exact year providers attested to MU. Hence, the sample dataset was constructed as a cross-section with multiple years of data rather than a longitudinal dataset. Nonetheless, the pooled logistic regressions with GEE have been shown to provide consistent estimates of effects similar to those obtained with the use of time-dependent Cox analyses [72, 73].

### Conclusion

The findings from this study may provide timely information on the merits of optimizing technology in low-resourced practice settings. Medicaid beneficiaries are traditionally low-income and vulnerable individuals with multiple comorbidities [74]. While many feared that disparities could be exacerbated by the use of advanced EHR functions in the presence of existing “digital divide “, meaningful use of EHRs can yield health benefits for economically disadvantaged communities that often are prone to experience non-optimal health outcomes [75]. Policies regarding increasing adoption and meaningful use of EHRs have typically focused on providing financial support to providers. While this is an important factor for many providers, particularly Medicaid providers, the findings for this study suggest that policy makers and healthcare providers should not ignore the contribution of county-level factors in the diffusion of EHR technologies among physician practices. Indeed, these findings provide important insights about EHR diffusion in places where traditionally underserved populations live. But this county-perspective is particularly important because of the potential for health IT to enable public health monitoring and population health management that might benefit individuals beyond the patients treated by the Medicaid providers [76, 77].

This study is consistent with the Healthy People 2030 Health IT objectives of the ONC to increase the proportion of physician practices that have necessary information electronically available at the point of care, including those in geographically disadvantaged areas [10]. It is also consistent with the ONC’s national priority that the benefits of meaningful use of EHRs accrue to all patient populations as disparities in home broadband service by geographic location continue to persist [10, 78]. Research is needed to further investigate geographic characteristics in which EHR diffusion initiatives can be leveraged to optimize technology use among underserved populations.

## References

[1] Battisti G, Stoneman P. Inter- and Intra-firm effects in the diffusion of new process technology. Res Pol. 2003;32: 1641–1655.

[2] Ajami S, Bagheri-Tadi T. Barriers for adopting electronic health records (EHRs) by physicians. Acta Inform Med. 2013;21(2): 129–134. doi: 10.5455/aim.2013.21.129-134 24058254 PMC3766548

[3] Dranove D, Forman C, Goldfarb A, Greenstein S. The trillion dollar conundrum: Complementarities and health information technology. Am Eco J: Econ Pol. 2014;6(4): 239–270.

[4] Bureau of Labor Statistics. Occupational Employment and Wages. 2021. Available from: https://www.bls.gov/oes/current/oes291215.htm.

[5] McCullough JS, Parente S, Town R. Health information technology and patient outcomes: The role of information and labor coordination. Rand J Econ. 2016;47(1): 207–236.

[6] The American Recovery and Reinvestment Act of 2009. Public Law. 2009;111(5): 5–30.

[7] Blumenthal D, Tavenner M. The "meaningful use" regulation for electronic health records. N Engl J Med. 2010;363(6): 501–504. doi: 10.1056/NEJMp1006114 20647183

[8] Centers for Medicare and Medicaid Programs. Electronic Health Record Incentive Program; Final Rule. Fed. Regist. 2010; 75(144): 44314. 20677415

[9] Centers for Medicare and Medicaid Services (CMS). An Introduction to the Medicaid EHR Incentive Program for Eligible Professionals. Baltimore, MD: Centers for Medicare and Medicaid Services; 2014. Available from: https://www.cms.gov/regulations-and-guidance/legislation/ehrincentiveprograms/downloads/ehr_medicare_stg1_begguide.pdf.

[10] Office of the National Coordinator (ONC) for Health Information Technology. Federal health IT strategic pPlan 2020–2025.; 2021. Available from: https://www.healthit.gov/topic/2020-2025-federal-health-it-strategic-plan.

[11] Adler-Milstein J, DesRoches CM, Furukawa MF, Worzala C, Charles D, Kralovec P, et al. More than half of US hospitals have at least a basic EHR, but stage 2 criteria remain challenging for most. Health Aff (Millwood). 2014;33(9): 1664–1671. doi: 10.1377/hlthaff.2014.0453 25104826

[12] Adler-Milstein J, Furukawa MF, King J, Jha AK. Early results from the hospital Electronic Health Record Incentive Programs. Am J Manag Care. 2013;19(7): e273–284. 23919447

[13] Pérez-Stable EJ, Jean-Francois B, Aklin CF. Leveraging advances in technology to promote health equity. Med Care. 2019;57: S101–S103. doi: 10.1097/MLR.0000000000001112 31095045

[14] Weiss D, Rydland HT, Øversveen E, Jensen MR, Solhaug S, Krokstad S. Innovative technologies and social inequalities in health: A scoping review of the literature. PLOS ONE. 2018;13(4): e0195447. doi: 10.1371/journal.pone.0195447 29614114 PMC5882163

[15] Aldrich HE, Pfeffer J. Environments of organizations. Annu Rev Sociol. 1976;2: 79–105.

[16] Pfeffer J, Salancik GR. The external control of organizations: A resource dependence perspective,. New York, NY.: Harper & Row; 1978.

[17] Scott WR, Davis GF. Organizations and organizing: rational, natural, and open system perspectives. Upper Saddle River, NJ: Pearson Prentice Hall; 2007.

[18] Kazley AS, Ozcan YA. Organizational and environmental determinants of hospital EMR adoption: a national study. J Med Syst. 2007;31(5): 375–384. doi: 10.1007/s10916-007-9079-7 17918691

[19] Menachemi N, Collum TH. Benefits and drawbacks of electronic health record systems. Risk Manag Healthc Policy. 2011;4: 47–55. doi: 10.2147/RMHP.S12985 22312227 PMC3270933

[20] Pfeffer J., Salancik GR. The external control of organizations: A resource dependence perspective (Stanford Business Classics). 1st ed. Stanford, CA: Stanford Business Classics; 2003.

[21] Trinh HQO’ConnorSJ. Helpful or harmful? The impact of strategic change on the performance of U.S. urban hospitals. Health Serv Res. 2002;37(1): 145–171. 11949918

[22] Zinn JS, Proenca J, Rosko MD. Organizational and environmental factors in hospital alliance membership and contract management: A resource-dependence perspective. J Healthc Manag. 1997;42(1): 67–86. 10164899

[23] Menachemi N, Powers TL, Brooks RG. Physician and practice characteristics associated with longitudinal increases in electronic health records adoption. J Health care Manag. 2011;56(3): 183–197. 21714373

[24] Ford EW, Menachemi N, Phillips MT. Predicting the adoption of electronic health records by physicians: when will health care be paperless? J Am Med Inform Assoc. 2006;13(1): 106–112. doi: 10.1197/jamia.M1913 16221936 PMC1380189

[25] Hsieh H-M., Bazzoli GJ. The impact of Medicaid disproportionate share hospital payment on the provision of hospital uncompensated care. Inq. 2012; 49(3): 254–267. 10.5034/inquiryjrnl_49.03.02 PMC3530404 23230705

[26] Scott WR. Organizations: Rational, Natural, and Open Systems. Englewood Cliffs, NJ.: Prentice‐Hall; 2003.

[27] Kissam S, Banger A, Dimitropoulos L, Thompson C. Barriers to meaningful use in Medicaid: analysis and recommendations. Rockville, MD: Agency for Healthcare Research and Quality. 2012. Available from: https://digital.ahrq.gov/sites/default/files/docs/citation/BarrierstoMeaningfulUseFinalReport.pdf.

[28] Yeager VA, Menachemi N, Savage GT, Ginter PM, Sen BP, Beitsch LM. Using resource dependency theory to measure the environment in health care organizational studies: a systematic review of the literature. Health Care Manag Rev. 2014;39(1): 50–65. doi: 10.1097/HMR.0b013e3182826624 23358132

[29] Or C, Tong E, Tan J, Chan S. Exploring factors affecting voluntary adoption of electronic medical records among physicians and clinical assistants of small or solo private general practice clinics. J Med Syst. 2018;42(7): 1–12. doi: 10.1007/s10916-018-0971-0 29845400

[30] Monestime JP, Freeman K, Alexandre PK. Provider participation in the Florida Medicaid Promoting Interoperability program: Practice characteristics, meaning use attestations, and incentive payments. Int J Med Inform. 2021;150: e104441. doi: 10.1016/j.ijmedinf.2021.104441 33823463

[31] Agency for Health Care Administration (AHCA). EHR Florida Medicaid Incentive Program Promoting Interoperability. Provider Participation Report 2011–2018. Tallahassee, FL: State of Florida, 2018. Available from: https://ahca.myflorida.com/medicaid/electronic-health-record-incentive-program

[32] Florida Office of Insurance Regulation (FOIR). Managed Care Enrollment Annual Reports [Internet]; 2018. Available from: https://floir.com/resources-and-reports/annual-report.

[33] Dept of Health and Human Services. Area Health Resource Files (AHRFs). Health Resources and Services Administration (HRSA). 2019. [Cited 2022 Jan 23] Available from: https://data.hrsa.gov/data/download.

[34] Agency for Health Care Administration (AHCA). Florida’s Rural Counties. https://www.floridahealth.gov/programs-and-services/community-health/rural-health/_documents/ruralcounties2012.pdf. 2012.

[35] Tarver WL, Menachemi N. Environmental market factors associated with electronic health record adoption among cancer hospitals. Health Care Manag Rev. 2018;43(4): 303–314. doi: 10.1097/HMR.0000000000000149 28225447

[36] Villalonga B, Raphael A. How Do Family Ownership, Control and Management Affect Firm Value? J Financ Econ. 2006;80: 385–417.

[37] Bloom N, Van Reenen J. Measuring and Explaining Management Practices across Firms and Countries. Quart J Econ. 2007;122: 1351–1408.

[38] Jung H-Y, Unruh MA, Kaushal R, Vest JR. Growth of new york physician participation in meaningful use of electronic health records was variable, 2011–12. Health Aff. 2015;34(6): 1035–1043. doi: 10.1377/hlthaff.2014.1189 26056210

[39] Raglan GB, Margolis B, Paulus RA, Schulkin J. Electronic health record adoption among obstetrician/gynecologists in the United States: physician practices and satisfaction. J Healthc Qual. 2014. 10.1111/jhq.12072. 28481842

[40] Menachemi N, Mazurenko O, Kazley AS, Diana ML, Ford EW. Market factors and electronic medical record adoption in medical practices. Health Care Manag Rev. 2012;37(1): 14–22. doi: 10.1097/HMR.0b013e3182352562 22016180

[41] Wasserman CO E. Department of Health Agency Standards for Reporting Data with Small Numbers. Seattle, WA: Washington State Dept of Health; 2018. Available from: https://doh.wa.gov/sites/default/files/legacy/Documents/1500//SmallNumbers.pdf?uid=625e058e8cdcf.

[42] Tiwari C, Beyer K, Rushton G. The impact of data suppression on local mortality rates: the case of CDC WONDER. Am J Public Health. 2014;104(8): 1386–1388. doi: 10.2105/AJPH.2014.301900 24922161 PMC4103252

[43] Cameron AC, Trivedi PK. Microeconometrics: methods and applications: Cambridge university press; 2005.

[44] Greene W. Fixed effects and bias due to the incidental parameters problem in the Tobit model. Econom Rev. 2004;23(2): 125–147.

[45] Wooldridge JM. Selection Corrections for panel data models under conditional mean: Developments in the econometrics of panel data. J Econom. 1995; 68(1): 115–132.

[46] Zeger SL, Liang KY, Albert PS. Models for longitudinal data: a generalized estimating equation approach. Biometrics. 1988;44(4): 1049–1060. 3233245

[47] Wooldridge JM. Distribution-free estimation of some nonlinear panel data models. J Econom. 1999;90(2): 77–97.

[48] Wooldridge JM. Introductory econometrics. A modern approach. Boston, MA: Cengage Learning; 2015.

[49] StataCorp. Stata statistical software: Release 16. College Station, TX: StataCorp LLC.; 2019.

[50] United States Department of Agriculture (USDA). Rural poverty and well-being. 2022. [Cited 2022 December 20. Available from: https://www.ers.usda.gov/topics/rural-economy-population/rural-poverty-well-being/.

[51] Casey MM, Moscovice I, McCullough J. Rural primary care practices and meaningful use of electronic health records: the role of regional extension centers. J Rural Health. 2014;30(3): 244–251. doi: 10.1111/jrh.12050 24118180

[52] Whitacre BE. Rural EMR adoption rates overtake those in urban areas. J Am Med Inform Assoc. 2015;22(2): 399–408. doi: 10.1093/jamia/ocu035 25665701 PMC8485927

[53] Office of the National Coordinator for Health Information Technology (ONC). Regional extension centers (RECs); 2018 [updated 2018 November 7]. Available from: https://www.healthit.gov/topic/regional-extension-centers-recs.

[54] Lynch K, Kendall M, Shanks K, Haque A, Jones E, Wanis MG, et al. The Health IT Regional Extension Center Program: evolution and lessons for health care transformation. Health serv res. 2014;49(1pt2): 421–437. doi: 10.1111/1475-6773.12140 24359032 PMC3925411

[55] Mostashari F, Tripathi M, Kendall M. A tale of two large community electronic health record extension projects. Health Aff. 2009;28(2): 345–356. doi: 10.1377/hlthaff.28.2.345 19275989

[56] Samuel CA. Area-level factors associated with electronic health record adoption and meaningful use in the Regional Extension Center Program. J Am Med Inform Assoc. 2014;21(6): 976–983. doi: 10.1136/amiajnl-2013-002347 24798687 PMC4215037

[57] DesRoches CM, Campbell EG, Rao SR, Donelan K, Ferris TG, Jha A, et al. Electronic health records in ambulatory care—a national survey of physicians. N Engl J Med. 2008;359(1): 50–60. doi: 10.1056/NEJMsa0802005 18565855

[58] Shields AE, Shin P, Leu MG, Levy DE, Betancourt RM, Hawkins D, et al. Adoption of health information technology in community health centers: results of a national survey. Health Aff. 2007;26(5): 1373–1383. doi: 10.1377/hlthaff.26.5.1373 17848448

[59] King J, Furukawa MF, Buntin MB. Geographic variation in ambulatory electronic health record adoption: implications for underserved communities. Health services research. 2013;48(6): 2037–2059. doi: 10.1111/1475-6773.12078 23800087 PMC3876400

[60] Jha AK, DesRoches CM, Shields AE, Miralles PD, Zheng J, Rosenbaum S, et al. Evidence of an Emerging Digital Divide Among Hospitals That Care For The Poor. Health Aff. 2009;28: w1160–w1170. doi: 10.1377/hlthaff.28.6.w1160 19858142

[61] Baker LC. Managed care and technology adoption in health care: Evidence from magnetic resonnance imaging. J Health Econ. 2001;20(3): 395–421. 11373838 10.1016/s0167-6296(01)00072-8

[62] Burt CW, Sisk JE. Which physicians and practices are using electronic medical records? Health Aff (Millwood). 2005;24(5): 1334–1343. doi: 10.1377/hlthaff.24.5.1334 16162581

[63] Menachemi N, Brooks RG. EHR and other IT adoption among physicians: results of a large-scale statewide analysis. J Healthc Inf Manag. 2006;20(3): 79–87. 16903665

[64] Baker LC. Managed care spillover effects. Annu Rev Public Health. 2003;24(1): 435–456. doi: 10.1146/annurev.publhealth.24.100901.141000 12471276

[65] Menachemi N, Shin DY, Ford EW, Yu F. Environmental factors and health information technology management strategy. Health Care Manag Rev. 2011;36(3): 275–285. doi: 10.1097/HMR.0b013e3182048e7e 21646886

[66] Abdolrasulnia M, Menachemi N, Shewchuk RM, Ginter PM, Duncan WJ, Brooks RG. Market effects on electronic health record adoption by physicians. Health Care Manag Rev. 2008;33(3): 243–252. doi: 10.1097/01.HMR.0000324904.19272.c2 18580304

[67] Duncan R. Characteristics of organizational environments and perceived environmental uncertainties. Adm Sci Q. 1972;17(3): 313–327.

[68] Kreiser MP, Marino LD. Analyzing the historical development of the environmental uncertainty construct. Manag Decis. 2002;40(9): 895–905.

[69] Health Florida. 2023. [Cited 2023 August 24]. Available from: https://www.flhealthcharts.gov/charts/default.aspx.

[70] Bureau of the Census. 2023. [Cited 2023 August 24]. Available from: www.census.gov.

[71] Mengiste SA, Antypas K, Johannessen MR, Klein J, Kazemi G. eHealth policy framework in Low and Lower Middle-Income Countries; a PRISMA systematic review and analysis. BMC Health Serv Res. 2023; 23:328. doi: 10.1186/s12913-023-09325-7 37005588 PMC10067308

[72] Ngwa JS, Cabral HJ, Cheng DM, Pencina MJ, Gagnon DR, LaValley MP, et al. A comparison of time dependent Cox regression, pooled logistic regression and cross sectional pooling with simulations and an application to the Framingham Heart Study. BMC Med Res Methodol. 2016;16(1):148. doi: 10.1186/s12874-016-0248-6 27809784 PMC5094095

[73] D’Agostino RB, Lee ML, Belanger AJ, Cupples LA, Anderson K, Kannel WB. Relation of pooled logistic regression to time dependent Cox regression analysis: the Framingham Heart Study. Stat Med. 1990;9(12): 1501–1515. doi: 10.1002/sim.4780091214 2281238

[74] Kaiser Permanente. State Health Facts/Providers and Service Use. 2022 [cited June 29, 2022]. Available from: https://www.kff.org/state-category/providers-service-use/.

[75] Jean-Francois B, Bailey Lash T, Dagher RK, Green Parker MC, Han SB, Lewis Johnson T. The potential for health information technology tools to reduce racial disparities in maternal morbidity and mortality. J Womens Health. 2021;30(2): 274–279. doi: 10.1089/jwh.2020.8889 33211604 PMC8020554

[76] Buck MD, Taverna J, Amirfar S, Stubbs-Dame R, Singer J. The Hub population health system: distributed ad hoc queries and alerts. JAMIA 2012; 19: 46–50 doi: 10.1136/amiajnl-2011-000322 22071531 PMC3392869

[77] Diamond CC, Mostashari F, Shirky C. Collecting an dsharing data for population health: a new paradigm. 2009. Health Aff. 2009;28(2): 454–466 doi: 10.1377/hlthaff.28.2.454 19276005

[78] Department of Health and Human Services (DHHS). Healthy people 2030: Health IT objectives; 2022. Available from: https://health.gov/healthypeople.

